# Nuts in the Prevention and Management of Type 2 Diabetes

**DOI:** 10.3390/nu15040878

**Published:** 2023-02-09

**Authors:** Stephanie K. Nishi, Effie Viguiliouk, Cyril W. C. Kendall, David J. A. Jenkins, Frank B. Hu, John L. Sievenpiper, Alessandro Atzeni, Anoop Misra, Jordi Salas-Salvadó

**Affiliations:** 1 Universitat Rovira i Virgili, Departament de Bioquímica i Biotecnologia, Unitat de Nutrició, 43201 Reus, Spain; 2 Institut d’Investigació Sanitària Pere Virgili (IISPV), 43201 Reus, Spain; 3 Centro de Investigación Biomédica en Red Fisiopatología de la Obesidad y la Nutrición (CIBEROBN), Institute of Health Carlos III, 28029 Madrid, Spain; 4 Toronto 3D (Diet, Digestive Tract and Disease) Knowledge Synthesis and Clinical Trials Unit, Toronto, ON M5C 2T2, Canada; 5 Clinical Nutrition and Risk Factor Modification Centre, St. Michael’s Hospital, Unity Health Toronto, Toronto, ON M5C 2T2, Canada; 6 Department of Nutritional Sciences, Temerty Faculty of Medicine, University of Toronto, Toronto, ON M5S 1A1, Canada; 7 College of Pharmacy and Nutrition, University of Saskatchewan, Saskatoon, SK S7N 5E5, Canada; 8 Department of Medicine, Temerty Faculty of Medicine, University of Toronto, Toronto, ON M5S 1A1, Canada; 9 Division of Endocrinology and Metabolism, Department of Medicine, St. Michael’s Hospital, Toronto, ON M5B 1W8, Canada; 10 Li Ka Shing Knowledge Institute, St. Michael’s Hospital, Toronto, ON M5B 1T8, Canada; 11 Department of Nutrition, Harvard T.H. Chan School of Public Health, Boston, MA 02115, USA; 12 Department of Epidemiology, Harvard T.H. Chan School of Public Health, Boston, MA 02115, USA; 13 Channing Division of Network Medicine, Department of Medicine, Brigham and Women’s Hospital and Harvard Medical School, Boston, MA 02115, USA; 14 Fortis C-DOC Hospital for Diabetes & Allied Sciences, New Delhi 110048, India; 15 National Diabetes, Obesity and Cholesterol Foundation, New Delhi 110016, India; 16 Diabetes Foundation (India), New Delhi 110070, India

**Keywords:** nuts, diabetes, glycemic control, insulin resistance

## Abstract

Diabetes is a continuously growing global concern affecting >10% of adults, which may be mitigated by modifiable lifestyle factors. Consumption of nuts and their inclusion in dietary patterns has been associated with a range of beneficial health outcomes. Diabetes guidelines recommend dietary patterns that incorporate nuts; however, specific recommendations related to nuts have been limited. This review considers the epidemiological and clinical evidence to date for the role of nut consumption as a dietary strategy for the prevention and management of type 2 diabetes (T2D) and related complications. Findings suggest nut consumption may have a potential role in the prevention and management of T2D, with mechanistic studies assessing nuts and individual nut-related nutritional constituents supporting this possibility. However, limited definitive evidence is available to date, and future studies are needed to elucidate better the impact of nuts on the prevention and management of T2D.

## 1. Introduction

Type 2 diabetes is one of the most globally challenging and prevalent metabolic disorders affecting an estimated 1 in 10 adults (10.5% of adults worldwide) [1]. Within the past 2 years, type 2 diabetes prevalence has risen by 16%, indicating an alarming growth rate [1]. Complications of diabetes, such as cardiovascular disease, chronic kidney disease, neuropathy, and retinopathy, and its high medical and other economic expenditures are a serious cause of concern [2]. Excluding mortality risks associated with COVID-19, approximately 12.2% of global adult deaths from all-cause are estimated to have occurred due to diabetes or its complications in 2021 [2]. Further, 10.6% of adults worldwide have impaired glucose tolerance, placing them at high risk for developing type 2 diabetes (T2D) [2].

Lifestyle changes, such as those related to nutrition, underpin a general approach to diabetes risk minimization and management. Current diabetes guidelines recommend dietary patterns, such as Mediterranean and vegetarian patterns, which encourage the consumption of nuts [3,4,5,6]. Nuts, represented by tree nuts (almonds, Brazil nuts, cashews, hazelnuts, macadamias, pecans, pine nuts, pistachios, walnuts) and peanuts (technically a legume, but sharing a similar nutritional and culinary profile to tree nuts, hence, their inclusion in the “nuts” classification), are nutrient-dense foods with complex matrices providing unsaturated fatty acids, plant-protein, non-sodium minerals, phenolic and other bioactive compounds [7,8,9].

In this narrative review, we summarize the human evidence currently available (“Where we are”) for the role of nuts in the prevention and management of T2D and discuss future directions (“Where we are going”) in terms of what questions may still need to be addressed and how research may address and inform any knowledge gaps. For this narrative review, a comprehensive search of PubMed and Cochrane databases through November 2022 for English language articles of epidemiological, clinical studies, and the latest reviews and meta-analyses assessing nut consumption (tree nuts and peanuts) and their components on T2D and related risk factors was conducted. The present article is not a systematic review; thus, some studies may not have been identified; further, the possibility of publication bias should be acknowledged. Nonetheless, the authors independently conducted literature searches, and these findings were further shared and discussed among an assembly of experts in the field of nut and health research.

## 2. Effect of Nut Consumption on Measures of Glucose Metabolism

Few epidemiological studies have assessed the association between nut consumption and markers of glycemic control. Table 1 summarizes epidemiological and clinical findings related to nut consumption and measures of glucose metabolism. To our knowledge, there is a lack of prospective cohort studies that have analyzed markers of glucose metabolism in individuals with or without diabetes. One prospective population-based study, conducted within the framework of the Tehran Lipid and Glucose study (TLGS), presented fasting serum glucose measures following a median 6.2-year follow-up across tertiles of nut consumption. At 6.2 years, findings showed lower fasting serum glucose levels in the highest tertile of nut consumption (nut intake: median 8.7 g/week, IQR, 5.3 to 15.8 g/week; fasting glucose: 4.7 ± 0.1 mmol/L) compared to the lowest tertile (nut intake: median 1.6 g/week, IQR, 0.7 to 2.8 g/week; fasting glucose: 5.3 ± 0.1 mmol/L) (*p* = 0.02) [10]. However, there appears to be a shortage of evidence in relation to other glucose-metabolism-related biomarkers and in individuals with diabetes. Cross-sectional studies have shown an association between nut consumption and markers of glucose/insulin homeostasis. One cross-sectional study assessing data from 16,784 American adults (51.8% women, aged ≥18 years) participating in the National Health and Nutrition Examination Survey (NHANES, 2005–2010) evaluated the association between nut intake and markers of glycemic control [11]. The authors observed that higher nut intake was associated with significantly lower levels of all diabetes-related biomarkers, including fasting blood glucose, plasma insulin, homeostasis model assessment-insulin resistance (HOMA-IR), HOMA-β, glycated hemoglobin (HbA1c), and oral glucose tolerance test (OGTT) (*p* < 0.001). Another cross-sectional study analyzed the association between the frequency of nut consumption and insulin resistance, measured by HOMA-IR, in 379,310 Koreans [12]. In this study, nut consumption ≥5 servings/week (where 1 serving = 15 g) compared to <1 serving/month was associated with lower HOMA-IR (odds ratio [OR]: 0.90; 95% confidence interval [CI] 0.86 to 0.94). This association was observed to be more prominent in women, participants with normal glycaemia, and younger age (<40 years).

When considering evidence from clinical trials, consumption of nuts alone and when added to high glycemic index (GI) foods show a lowering in postprandial glycemia when compared to consumption of high GI foods alone. Several acute trials have assessed the effect of almond intake on postprandial glycemia. In healthy individuals, the consumption of almonds with white bread was shown to significantly lower the postprandial area under the insulin concentration vs. time curve when compared to a high GI meal (instant mashed potatoes) (n = 15) [41] and significantly lower the glucose peak height when compared with white bread (n = 9) [27]. In another acute randomized crossover trial conducted in healthy participants (n = 100 with available data, n = 106 randomized), consumption of at least 10% of energy from raw almonds resulted in the mean area under the blood glucose response curve being significantly lowered when compared to consumption of biscuits [28]. Similar findings were shown for individuals at higher risk of diabetes. In an acute randomized five-arm crossover trial conducted in individuals with impaired glucose tolerance (n = 14), participants were randomized to consume whole almonds, almond butter, defatted almond flour, almond oil, or no almonds that were incorporated into a 75 g available carbohydrate-matched breakfast meal. Whole almonds significantly diminished the second meal and daylong blood glucose incremental area under the curve and elicited a greater second-meal insulin response [20]. Another acute randomized crossover trial conducted in individuals with good health (n = 12) and individuals with T2D (n = 7) showed consumption of 28 g of almonds with a test meal (bagel, juice, and butter) significantly reduced postprandial glycemia in participants with diabetes but not in participants without diabetes when compared to the test meal without almonds [21]. For pistachio intake, an acute trial conducted in healthy individuals (n = 10) showed consumption of pistachios alone and, when added to white bread at different doses (28 g, 56 g, 84 g), significantly lowered glycemic responses in comparison to white bread [42]. The addition of pistachios to other commonly consumed carbohydrate-rich foods (parboiled rice, pasta, potatoes) also resulted in reduced glycemic responses [42]. Similarly, in an acute trial conducted on individuals with metabolic syndrome (n = 20), the consumption of pistachios with white bread significantly lowered the glycemic response and increased insulin secretagogue levels when compared to white bread alone [22]. For mixed nuts, an acute trial conducted in individuals with good health (n = 14) and in individuals with T2D (n = 10) showed mixed nuts at three different doses significantly reduced the glycemic response in comparison to white bread. The addition of mixed nuts to white bread progressively reduced the glycemic response of the meal; however, in individuals with T2D, the reduction in glycemic response was half that seen in healthy individuals [23]. In another acute trial, adults with overweight/obesity (n = 54) were randomized to consume either mixed nuts or pretzels and showed pretzel consumption increased glucose and insulin, whereas, with mixed nuts, no elevation was detected at 60 min post snack consumption [24]. For peanut intake, an acute trial conducted in men with overweight/obesity (n = 65) who consumed a test meal of a shake containing conventional peanuts, high-oleic peanuts, or a control biscuit showed a quicker return of insulin to basal concentrations after consumption of the shakes containing conventional peanuts and high-oleic peanuts [25].

Several systematic reviews and meta-analyses (SRMAs) of randomized controlled trials (RCTs) with a duration of at least 3 weeks have been conducted assessing the effect of a tree nut(s) on markers of glycemic control in people with different health statuses (the effect of tree nuts on markers of glycemic control in people with diabetes is discussed in Section 5.2). In 2014, SRMA of 49 RCTs (n = 2226) was conducted to assess the effect of tree nuts on metabolic syndrome criteria, including fasting glucose. Twenty-six trials were included (n = 1360) for fasting glucose, which showed tree nuts significantly lowered fasting glucose compared with the controls (mean difference [MD] = −0.08 mmol/L; 95% confidence interval [CI] −0.16 to −0.01 mmol/L) [43]. In 2018, a network meta-analysis of RCTs assessed the effect of different food groups on intermediate disease markers in adults, including fasting glucose, HbA1c, and HOMA-IR [44]. The results showed nuts were more effective at reducing fasting blood glucose when compared to red meat and fruits and vegetables, as well as HOMA-IR when compared to eggs and dairy. No significant effects were shown for HbA1c. In 2019, another SRMA of 40 RCTs (n = 2832) was conducted to assess the effect of tree nut or peanut intake in adults on glycemic control, including fasting glucose, fasting insulin, HbA1c, and HOMA-IR. Nut intake showed a significant lowering in fasting insulin (28 RCTs; weighted mean difference [WMD]: −0.40 μIU/mL; 95% CI: −0.73, −0.07 μIU/mL; I^2^ = 49.4%) and HOMA-IR (19 RCTs; WMD: −0.23; 95% CI: −0.40, −0.06; I^2^ = 51.7%), with no significant effect on fasting glucose or HbA1c [40]. Subgroup analysis by nut type showed a significant reduction in fasting blood glucose with pistachio consumption compared with the control (WMD: −5.18 mg/dL; 95% CI: −8.76, −1.60 mg/dL; I^2^ = 67%). This was supported by another SRMA of RCTs published in 2020, assessing the effect of pistachio intake on glycemic control in individuals with different health statuses (type 2 diabetes, prediabetes, and metabolic syndrome), which showed a significant reduction in fasting glucose and HOMA-IR but not HbA1c or fasting insulin [34]. Tindall et al. also identified a small number of studies that measured outcomes related to insulin production and HOMA-β cell function (7 studies), glucose concentrations after a 75-g OGTT (5 studies), insulin concentrations after a 75-g OGTT (2 studies), insulin sensitivity (3 studies) and short-term glucose control (2 studies) [40]. Due to a limited number of trials that measured these endpoints and the heterogeneity in the measurements, a meta-analysis was not performed. These studies showed no impact of nut intake on outcomes related to insulin concentrations after a 75 g OGTT and insulin sensitivity, whereas there were mixed findings for outcomes related to insulin production and HOMA-β cell function, glucose concentrations after a 75 g OGTT, and short-term glucose control [40]. Several other SRMAs of RCTs have been conducted between 2020 and 2022, assessing the effect of a specific nut type and/or the effect of nuts in a specific group of people. These SRMAs assessed the effect of different types of nuts in healthy adults with overweight/obesity (10 RCTs) [45], walnuts in middle-aged and older adults (17 RCTs) [46] and individuals with different health statuses (16 RCTs) [47], cashews (6 RCTs) [48], peanuts (11 RCTs) [49], and 2 SRMAs investigating almonds in individuals with different health statuses (24 RCTs [50], 15 RCTs [51]), all of which showed no impact on markers of glycemic status.

Since the publication of the above-mentioned SRMAs, more recent RCTs in people without diabetes have been published. In a 6-month RCT, 107 individuals who were overweight and at moderate or high risk of T2D were randomized to either an energy-restricted diet, including 70 g/d of peanuts or an energy-restricted low-fat diet, which showed no significant differences between groups in regard to HbA1c, fasting glucose, fasting insulin, 2 h glucose, and HOMA-IR [52]. In an 8-week RCT, 40 women were randomized to an energy-restricted diet without nuts or to an energy-restricted diet containing 45 g/d of nuts (15 g of Brazil nuts + 30g of cashew nuts), which also showed no significant differences in markers of glycemic status [53].

## 3. Nuts and Diabetes Prevention

Table 1 summarizes epidemiological and clinical findings related to nut consumption and diabetes prevention.

### 3.1. Epidemiological Evidence

Epidemiological studies conducted to date have shown inconsistent and inconclusive evidence related to nut consumption and the incidence of T2D. A number of SRMAs involving cross-sectional or prospective cohort studies have been published investigating associations between the frequency of nut consumption and the prevalence and/or the incidence of T2D risk. Most have not reported a significant association when comparing the highest to the lowest categories of nut consumption, nor were dose-response relationships observed [54,55,56,57,58]. Only one of these meta-analyses of prospective cohort studies showed a significant inverse association with the risk of T2D [59]. However, a key limitation is that most of these SRMAs included studies combining nuts with other plant foods as the exposure (i.e., peas, seeds, or legumes) and, therefore, the associations cannot be extrapolated specifically to the possible role of nuts per se [55,58,59]. Additionally, in some of the observational studies, the associations were adjusted for body weight or BMI, a potential mediator of the associations [11] and, therefore, possibly attenuating an association.

In 2021, an updated SRMA of cross-sectional (n = 3) and prospective (n = 5) studies, including only those with nuts alone as an exposure, was published [13]. The included studies were conducted in the United States (5 studies), Europe (3 studies), and Asia (1 study). Findings from the meta-analyses of the cross-sectional studies (n = 72,559; 7559 cases of T2D) showed no significant association with diabetes prevalence when the highest compared to the lowest categories of total nut consumption was assessed (OR: 0.91; 95% CI: 0.83 to 1.01). When the prospective cohort studies were analyzed, no associations with risk of T2D were observed with consumption of total nuts (relative risk [RR]: 1.04: 95% CI: 0.94 to 1.15), tree nuts (RR: 0.98; 95% CI 0.87 to 1.11), or peanuts (RR: 0.95; 95% CI: 0.87 to 1.04). When peanut butter consumption was specifically assessed, it was shown to be inversely associated with T2D incidence (RR: 0.87; 95% CI: 0.77 to 0.98). Furthermore, there was no evidence of a linear dose-response or nonlinear dose-response gradient for the total nut or peanut consumption in prospective cohort studies. Of note, these analyses were adjusted for baseline BMI. Across all nut exposures evaluated, the certainty of the evidence was considered to be very low. The reduction in the risk of T2D seen in sensitivity analyses of this meta-analysis suggested that weight loss or decreased weight might mediate the reduction in risk, although appropriate statistical mediation analyses using repeated assessments are needed to confirm this assumption. It is important to highlight that in relation to the type of tree nuts, only one cross-sectional (n = 27,563) [15] and one prospective cohort (n = 137,956) study [14] had analyzed the association between the frequency of walnut consumption and T2D risk, reporting in both cases an inverse association with the prevalence and incidence of T2D, respectively. Of note, the largest prospective cohort study involving American adults participating in the Nurses’ Health Study (NHS; 58,063 women aged 52–77 [1998–2008]) and NHS II (79,893 women aged 35–52 years [1999–2009]), free of diabetes, cardiovascular disease, or cancer at baseline, observed that consumption of ≥2 servings/week (where 1 serving = 28 g) of walnuts had a 24% (95% CI: 6–38%) lower risk of developing T2D than those that never or almost never consumed walnuts after adjustment for baseline BMI [14].

Following the publication of the 2021 SRMA by Becerra-Tomás and colleagues, two additional cross-sectional studies from Italy and Spain involving community-dwelling adults have been published. Both studies reported no association between nut consumption and the prevalence of T2D [16,17].

In view of the studies published to date, new research is needed that prospectively assesses differences in existing cohorts. Moreover, dose-response analyses are warranted in the future to determine the total amount of nuts associated with possible diabetes-related health benefits to better inform guidelines and practice.

### 3.2. Clinical Trial Evidence

Unfortunately, to date, no clinical trials have been conducted with the primary aim of testing the ability of nut supplementation to reduce or prevent the incidence of diabetes, probably because such types of trials are very expensive and difficult to perform. However, data is available in relation to a secondary analysis conducted in the context of the PREDIMED (PREvención con DIeta MEDiterránea) study, a randomized controlled trial aiming to assess the effect of a Mediterranean diet supplemented with virgin olive oil or nuts in comparison to a low-fat diet on primary prevention of cardiovascular disease [31].

A sub-analysis of this RCT conducted in participants from one of the 23 recruiting study centers (located in Reus, Spain) reported a beneficial effect of the Mediterranean diet enriched with 30 g/day of tree nuts (walnuts, almonds, and hazelnuts) on T2D prevention [31]. Results from the PREDIMED trial as a whole showed a non-significant decrease in the incidence of T2D when compared to participants in the group receiving the low-fat dietary advice [32,33]. It is important to recognize that due to the study design, it is not possible to quantify the beneficial effects secondary to the Mediterranean diet intervention or the nuts that participants consumed throughout the trial.

## 4. Nuts and Diabetes Management

Table 1 also summarizes epidemiological and clinical findings related to nut consumption and diabetes management.

### 4.1. Epidemiological Evidence

There is a lack of epidemiological evidence for the role of nut consumption in individuals with T2D for glucose control and the management of complications.

Of the available evidence, one prospective analysis including 16,217 men and women, from the Health Professionals Follow-up Study (HPFS, 1986–2014) and NHS (1980–2014), respectively, with diabetes mellitus at baseline or diagnosed during follow-up, showed that higher total nut consumption was associated with a lower risk of cardiovascular disease (CVD) incidence and mortality [18]. Specifically, for participants who consumed ≥5 servings of total nuts per week (1 serving = 28 g), compared to those who consumed <1 serving per month, multivariate-adjusted hazard ratios (HR; 95% CIs), showed reductions in total CVD incidence (HR = 0.83; 95% CI: 0.71–0.98; *p* trend = 0.01), coronary heart disease incidence (HR = 0.80; 95% CI: 0.67–0.96; *p* trend = 0.005), CVD mortality (HR = 0.66; 95% CI: 0.52–0.84; *p* trend < 0.001), and all-cause mortality (HR = 0.69; 95% CI: 0.61–0.77; *p* trend < 0.001). For specific types of nuts, higher tree nut consumption was associated with a lower risk of total CVD, coronary heart disease incidence, and mortality because of CVD, cancer, and all causes, whereas peanut consumption was associated with lower all-cause mortality only (all *p* trend <0.001). This study showed that higher consumption of nuts, especially tree nuts, may be associated with lower CVD incidence and mortality among participants with T2D.

A SRMA of four prospective cohort studies (n = 202,751) assessed the relationship of nut consumption with diabetes-related mortality, indicating higher nut intake to be associated with reduced risk of mortality from diabetes compared to the lowest intake [19]. A similar response was observed in the dose-response analysis, with a 39% reduction in the relative risk of diabetes mortality being observed with a one-serving/day (1 serving = 28 g) increase in nut consumption. Based on the findings of this SRMA and the assumption that the associations observed between nut consumption and diabetes mortality are causal, the authors estimated that for the regions assessed (i.e., North and South America, Europe, Southeast Asia, and Western Pacific), 139,000 deaths due to diabetes may be attributed to a nut intake below 20 g/day.

### 4.2. Clinical Trial Evidence

In clinical trials, consumption of nuts alone and when added to high GI foods show a lowering in postprandial glycemia when compared to the high GI food alone in people with diabetes. In an acute trial conducted in healthy individuals (n = 14) and in individuals with T2D (n = 10), mixed nuts at three different doses significantly reduced the glycemic response in comparison to white bread [23]. As previously noted, this trial also showed that adding mixed nuts to white bread progressively reduced the glycemic response of the meal. However, in individuals with T2D, the reduction in glycemic response was half that seen in healthy individuals. In a randomized crossover trial, the acute effects of almond intake were assessed in men with T2D (n = 7) randomized to consume a control (white bread, butter, cheese) and a test (white bread, almonds) meal. The test meal was found to be associated with lower postprandial glycemia and insulinemia, and an increased estimated glucose metabolic clearance rate [29]. Almonds were also assessed in another acute randomized controlled trial involving participants with (n = 7) and without (n = 12) diabetes [21]. Findings showed consumption of 28 g of almonds with a test meal, composed of a bagel, juice, and butter, significantly reduced postprandial glycemia in participants with diabetes but not those without diabetes when compared to the test meal without almonds.

Several SRMAs of RCTs have been conducted assessing the effect of tree nut(s) on markers of glycemic control in people with diabetes. In 2014, an SRMA of 12 RCTs assessing the effect of tree nuts on glycemic control in people with diabetes (n = 240) showed a significant lowering in fasting glucose (8 comparisons, MD = −0.15 mmol/L; 95% CI: −0.27, −0.02 mmol/L; I^2^ = 35%) and HbA1c (8 comparisons, MD = −0.07%: 95% CI: −0.10, −0.03%; I^2^ = 37%), with no significant effect on fasting insulin or HOMA-IR [35]. A 2019 SRMA of 40 RCTs (n = 2832) assessing the effect of tree nut or peanut intake in people with and without diabetes showed no significant effect on fasting glucose or HbA1c, and subgroup analyses by diabetes status showed no deviation from the main findings for either outcome [40]. There were a few differences in the inclusion/exclusion criteria between the 2014 and 2019 SRMAs. The 2019 SRMA [40] included trials using nut oil or peanuts as a treatment arm, non-isocaloric comparison arms, and studies published only in English. The 2014 SRMA [35] included only studies using whole tree nuts as the treatment arm, isocaloric comparison arms, and included studies that were not published in English. These differences may explain the discrepancy in findings. A more recent SRMA of RCTs published in 2021 (15 RCTs) assessed the effect of tree nuts on markers of glycemic control in individuals with T2D and showed no significant impact on fasting glucose, HbA1c, or postprandial glucose levels; however, the analysis only included RCTs with a follow-up period of 3 months or less [30]. Between 2020 and 2022 (present day), several other SRMAs of RCTs have been conducted assessing the effect of a specific nut type on markers of glycemic control in people with T2D. Two SRMA’s assessed the effect of almond intake; the first SRMA (8 RCTs) showed a significant lowering in HbA1c but no impact on fasting glucose, insulin, or HOMA-IR [36], whereas the second SRMA (9 RCTs) showed no impact on markers of glycemic control, including HbA1c, fasting glucose, and insulin [37]. Another SRMA assessed the effect of pistachio intake in individuals with T2D, prediabetes, and metabolic syndrome (6 RCTs), which showed a significant lowering in fasting glucose and HOMA-IR, but not HbA1c or fasting insulin [34].

Since the publication of the above-mentioned SRMAs, more recent RCTs assessing the effect of nut consumption on glycemic control in people with diabetes have been published. In a 3-month RCT, 45 people with T2D were randomized to either an almond-based, low-carbohydrate diet group or a low-fat diet group. After 3 months, individuals in the almond-based, low carbohydrate diet group showed a significant improvement in HbA1c [38]. In another 3-month RCT, 204 individuals with stable coronary artery disease (~32% of which had diabetes) were randomized to one of three groups: a healthy diet, a healthy diet plus 30 g/d of pecans or a healthy diet plus 30 mL/d of EVOO. After 12-weeks there were no significant differences between groups in regard to fasting glucose, HbA1c, fasting insulin or HOMA-IR [39].

## 5. Possible Mechanisms of Action of Nuts in Diabetes Prevention and Management

While the possible protective role of nuts in diabetes prevention and management remains to be established with greater certainty, there is potential for a beneficial impact given the unique nutritional composition of nuts and the direct and indirect evidence to date relating relevant dietary constituents with diabetes prevention and management.

**Figure 1:**
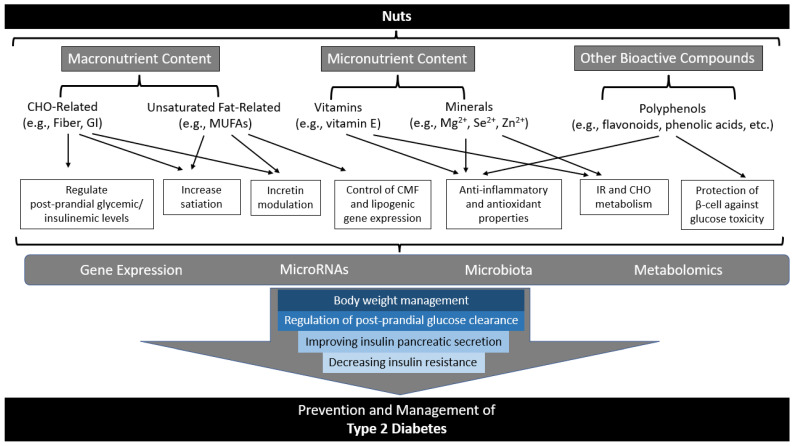
Summary of potential mechanisms of action for the role nuts may play in diabetes prevention and management. Adapted with permission from Ref. [60]. 2017, Hernández-Alonso et al. Abbreviations: CHO, carbohydrate; CMF, cellular membrane fluidity; GI, glycemic index; IR, insulin resistance; MUFAs, monounsaturated fatty acids; RNA, ribonucleic acid.

There are several proposed and speculative modulatory effects of the constituents of nuts in the prevention and management of T2D that may act synergistically (summarized in Figure 1).

Macronutrients, micronutrients, and other bioactive compounds found in nuts have been suggested to play a role in the regulation of postprandial glycemic and insulinemic levels. Furthermore, body weight management, control of cellular membrane fluidity and lipogenic gene expression, anti-inflammatory and antioxidant properties, and protection of β-cells against glucose toxicity and subsequent impacts on gene expression, microRNAs, and microbiota/metabolomics leading to regulation of postprandial glucose clearance, improving pancreatic insulin secretion, and decreasing insulin resistance have also been implicated with nut consumption or related factors. The following will briefly summarize available and relevant direct and indirect evidence for possible mechanisms for the impact of nut consumption on T2D related to macronutrients, micronutrients, other bioactive compounds, and resulting cellular and molecular mechanisms.

### 5.1. Related to Macronutrient Composition of Nuts

#### 5.1.1. Low Glycemic Index and Fiber

Nuts contain low amounts of available carbohydrates, meaning they do not contribute significantly to postprandial glycemia [61,62]. However, when nuts are added to foods with a high available carbohydrate, they demonstrate a dose-dependent reduction in the glycemic index or relative glycemic response of the composite meal [27,42]. This is thought to be due to their fat and protein content, which are a source of additional energy when added to food with highly available carbohydrates [27,61]. Several studies conducted around 20 years ago demonstrated that an increase in energy density from high-fat, protein, and/or high-fiber containing foods decreases gastric emptying [27,42,63,64,65]. Therefore, as the dose of nuts is increased, the rate of gastric emptying decreases, which may increase feelings of satiety and would decrease the postprandial glycemic response [27].

Nuts are also a source of dietary fiber [8,9]. Soluble fiber has been shown to increase the viscosity of intestinal contents and slow down the absorption of nutrients in the gastrointestinal tract [66]. Consumption of meals/foods containing soluble fiber have been shown to lower postprandial glycemia [66].

Fiber has also been shown to be resistant to digestion by enzymes in the small intestine and, as a result, susceptible to fermentation by bacteria in the colon, which leads to the production of short-chain fatty acids (SCFA) [66]. SCFAs have been shown to reduce hepatic glucose output and stimulate the secretion of the incretin hormone glucagon-like peptide 1 (GLP-1) [67,68]. GLP-1, as well as other incretins such as gastric inhibitory polypeptide (GIP), promote the proliferation of beta-cells and their secretion of insulin, which favors the maintenance of blood glucose levels [69]. As such, the consumption of nuts may slow the absorption of carbohydrates and stimulate incretin secretion, which can positively impact glucose homeostasis.

Furthermore, repeated decreases in postprandial glucose peaks, such as that observed with nut consumption, have been hypothesized to contribute to decreased inflammation, oxidation processes, and mitochondrial toxicity, further contributing to reductions in the risk of diabetes [60].

#### 5.1.2. Fatty Acids: Unsaturated vs. Saturated

Nuts have a high unsaturated fat content [8,9]. Substitution of carbohydrates or saturated fats (SFA) with unsaturated fats may be responsible for improvements in insulin sensitivity [40,70]. This was supported by an SRMA of 102 RCTs, which showed replacing carbohydrates or SFAs with monounsaturated or polyunsaturated fatty acids (MUFAs or PUFAs, respectively) improved markers of glycemic control, including HbA1c and HOMA-IR [70,71]. It should be noted that evidence involving the study of PUFAs tended to include a combination of these fatty acids, including eicosapentaenoic acid (EPA) and docosahexaenoic acid (DHA) along with alpha-linolenic acid (ALA); whereas, the PUFA found in nuts is ALA. Accordingly, additional investigation specific to ALA would better elucidate whether the PUFA content of nuts have the observed beneficial effects observed across overall PUFAs.

The quality of dietary fat can affect cell membrane composition and function, including membrane fluidity, insulin receptor binding/affinity, as well as facilitating the movement of the glucose receptor to the cell surface, which in turn can affect insulin sensitivity [72,73,74,75].

Dietary fat quality may also be involved in regulating gene expression and enzyme activity [72,76]. A diet high in unsaturated fatty acids, in particular, long-chain omega-6 and omega-3 polyunsaturated fatty acids, has been shown to lead to the suppression of lipogenic genes (genes of lipid synthesis) and induction of genes involved with fatty acid oxidation, which may reduce hepatic insulin resistance. Saturated fat and monounsaturated fat, on the other hand, do not appear to impact these same mechanisms. This was supported by a recent SRMA of 30 RCTs assessing the effect of omega-3 fatty acid supplementation on several cardiometabolic markers in people with T2D, which showed a significant lowering in HbA1c, fasting glucose and HOMA-IR [77].

Unsaturated fatty acids from nuts may also stimulate the secretion of GLP-1, which stimulates the secretion of insulin from beta-cells and promotes the proliferation of beta-cells and, therefore, improves beta-cell efficiency [70,78,79].

### 5.2. Related to Micronutrients and Other Bioactive Components of Nuts

A number of minerals, vitamins, and other bioactive components, which may be found within the nutritional composition of nuts, have been suggested to be protective against T2D and beneficial in the management of related complications.

#### 5.2.1. Vitamins and Minerals

Nuts, depending on the type, contain relatively high amounts of vitamin E, magnesium, and selenium, among other nutrients [7,8,9]. While direct evidence does not appear to be currently available for the impact of the content of micronutrients from nut consumption specifically on the risk and management of T2D, there is evidence from epidemiological and oral micronutrient supplementation studies suggesting nutrients that are found in relatively high amounts in nuts may be beneficial.

Evidence from multiple SRMAs has supported the association of specific vitamins and minerals, analogous to those found in nuts, with markers of glycemic control and prevention of T2D [80,81,82,83,84,85]. When oral supplementation of antioxidant vitamins and minerals (such as those found in nuts: vitamin E, selenium, and zinc) were assessed in a SRMA of RCTs considering people with T2D, supplementation of zinc (30 to 660 mg/day) and vitamin E (200 to 800 IU/day) reduced HbA1c, and zinc reduced fasting blood sugar. None of the nut-associated micronutrient supplements were effective in the reduction of insulin, HOMA-IR, or HOMA-B, and all evidence was considered to be low certainty [85].

Magnesium has also been associated with beneficial effects on glycemic control. Imbalances in magnesium status, specifically hypomagnesemia, have been shown to inhibit glucose transporter type 4 translocation, increase insulin resistance, and affect lipid metabolism, oxidative stress, and the antioxidant system of endothelial cells [82]. A SRMA of 12 observational studies showed significantly lower circulating magnesium levels in people with prediabetes compared to individuals with good health [83]. Further, a 100 mg/day increase in oral magnesium intake has been associated with a 15% reduction in T2D risk (SRMA prospective cohort studies, n = 286,668). For perspective, a 100 mg amount of magnesium is approximately equivalent to the magnesium content in about ¼ cup of nuts, depending on nut type [8,9]. When considering individuals with T2D, oral magnesium supplementation, equivalent to just over ½ cup of nuts, significantly improved glycemic control indicators, including HbA1c, IL, C-peptide, HOMA-IR, and HOMA-B and insignificantly decreased fasting blood glucose [86]. However, the impact of magnesium when consumed as a constituent of nuts may respond differently within the body compared to an oral magnesium supplement and further investigation may shed light on the possible role magnesium and/or other micronutrients from the consumption of nuts may have in the prevention and management of type 2 diabetes.

#### 5.2.2. Phenolics and Other Bioactive Compounds

Nuts are composed of a matrix of other important bioactive compounds, including polyphenols of various types (e.g., flavonoids, phenolic acids, stilbenes, lignans, other polyphenols) and concentrations (e.g., 126 to 1576 mg total polyphenols per 100 g nuts) [87,88,89]. There have been a number of investigations into the polyphenol characteristics of nuts and, independently, a number of studies have assessed the role of polyphenols in diabetes progression and management. For example, polyphenols may improve HbA1c and insulin resistance, in addition to having anti-inflammatory and antioxidant properties such as superoxide dismutase (SOD)-like activity, 1,1-diphenyl-2-picrylhydrazyl (DPPH), and radical scavenging activity. In these ways, various polyphenols may lower the risk of developing diabetes and its complications. However, there is limited evidence confirming the role of polyphenols from nuts in glycemic control, insulin sensitivity, and ultimately in the prevention and management of diabetes [87,90,91]. Polyphenols in nuts may also be protective against diabetes by modifying the gut microbiota (discussed further in Section 5.4.1). Currently, nuts appear to provide only a small percentage of polyphenols in the diet based on cohort data and global average nut intake levels [88,89,92]. Yet, consumption of approximately 50 g/day of nuts could provide the polyphenol dose observed with reduced T2D incidence [88].

### 5.3. Related to Body Weight and Adiposity

Approximately 60% to 90% of T2D has been attributable to obesity or weight gain; moreover, elevated weight can increase the risk of complications and comorbidities in people with diabetes [93,94] While nuts appear to be relatively high in calculated total calories and fat, their consumption has not been associated with weight gain nor an increased risk of overweight or obesity [95]. Conversely, despite their high energy density, a SRMA of six prospective cohort studies (n = 569,910) and 86 RCTs (n = 5873) indicated nut intake was associated with lower incidence of overweight/obesity (RR 0.93; 95% CI: 0.88 to 0.98), had no effect on body weight, and meta-regression showed higher nut consumption to be related to reductions in body weight and body fat [95]. Furthermore, adiposity factors (i.e., body mass index (BMI) and waist circumference) have been shown to play a role in mediating the association between nut consumption and markers of glycemic control (i.e., fasting blood glucose, plasma insulin, HOMA-IR, HbA1c, and OGTT) suggesting a potential mechanism for the prevention of diabetes risk [11].

### 5.4. Related to Cellular and Molecular Mechanisms of Nuts

As briefly noted in Section 3.1 and Section 3.2, the nutrient composition and bioactive compounds contained in nuts may play a role in preventing and managing diabetes through different cellular and molecular mechanisms, including the modulation of gut microbiota, modifying gene expression, or mediating gene expression through microRNAs (miRNAs). The following discusses evidence available explicitly related to nuts and these aspects.

#### 5.4.1. Gut Microbiota

Within the complex nutrient matrix of nuts, some of the components, such as fiber and polyphenols, can reach the colon intact and interact with the gut microbial population changing its composition and function [66]. The microbial colonic fermentation of undigested fiber and other nutrients from nuts can lead to the production of metabolites, such as SCFA (e.g., butyrate and propionate), with well-demonstrated positive effects for gut microbial homeostasis and may serve as a prebiotic [96,97].

SCFAs can induce their beneficial effects on glucose homeostasis by reducing gut motility and appetite stimulating the expression of peptide YY via the G-protein-coupled receptors (Gpr41 and Gpr43) [98]. Additionally, SCFAs may activate Gpr41 and Gpr43 on L-cells subsequently triggering the secretion of GLP-1, which improves glucose homeostasis by increasing the secretion of insulin and decreasing the secretion of glucagon. The activation of Gpr43 inhibits insulin signalling in adipocytes and fat accumulation in adipose tissue. Butyrate and propionate promote intestinal gluconeogenesis, reducing the risk of T2D. Butyrate also suppresses the action of histone deacetylase (HDAC), which induces insulin resistance by acting in different molecular pathways [99].

Nuts are also rich in polyphenols [87,88,89], and undigested polyphenols are thought to exert a prebiotic effect by stimulating the growth and activity of some bacteria, such as Bifidobacteria, in the digestive tract [100]. Increased levels of fecal Bifidobacteria have been associated with improved glucose tolerance and diminished inflammatory markers such as the interleukins IL-6, IL-1α and IL-1β, tumor necrosis factor α, and monocyte chemoattractant protein-1 [101].

Considering specific nut types, the effects of almonds on gut microbiota, glycometabolism, and inflammatory parameters in individuals with T2D have been explored in a systematic review conducted by Ojo et al. [36]. The results suggest that an almond-based diet could promote the growth of SCFA-producing bacteria in the gut. Walnuts are also rich in polyphenols and ellagitannins, which are metabolized by intestinal bacteria into urolithins. It has been shown that walnut supplementation, even if short-term, can impact the metabolism of ellagitannins to urolithins via gut microbiota by increasing the production of SCFA (such as acetate, butyrate, and propionate) [102]. This may impact the risk of T2D, as it has been observed that propionate can reduce serum cholesterol and improve insulin resistance, as well as promote satiety [103].

#### 5.4.2. Gene Expressions

Some nut components or their metabolites may act at the cellular level, modifying gene expression. Few studies have analyzed the impact of nut consumption on changes in gene expression in cells or tissues related to proteins that have important potential effects on carbohydrate metabolism, insulin resistance, or adiposity. The crossover EPIRDERM Study assessed the effect of pistachio intake (57 g/d for 4 months) versus a nut-free control diet on insulin resistance and T2D in participants with prediabetes (n = 54) showing changes in peripheral leukocyte gene expression and cellular glucose update [78]. Gene expression data showed that pistachio consumption, compared to control, significantly decreased the expression of interleukin-6 and resistin. Moreover, pistachio intake was shown to facilitate glucose transporter gene expression as assessed by SLC2A3 and SLC2A4 which showed different patterns. For instance, SLC2A4 appeared to be significantly increased in the control compared to pistachio phases. The percentage of change in cellular glucose transport activity also differed between the pistachio and control groups. Similarly, a significantly increased SLC2A4 protein expression on the surface of lymphocytes has been described in both individuals with diabetes and impaired glucose metabolism [104]. Consistent with this, attenuation in the expression of glucose transporters, with pistachio consumption leukocytes, was observed to be significantly expressed in T2D. Therefore, these results suggest a potential mechanism by which pistachios could lead to an improved systemic inflammatory profile increasing insulin sensitivity, as has been observed in the EPIRDERM study.

In a clinical trial conducted in 24 healthy participants, the consumption of hazelnuts (40g/d for 6 weeks) did not lead to weight gain, possibly due to the improvement of the body’s antioxidant capacity by the upregulation of genes [codifying superoxide dismutase 1 (SOD1), catalase (CAT), macrophage migration inhibitory factor (MIF), peroxisome proliferator-activated receptor gamma (PPAR-ɣ), vitamin D receptor (VDR) and methylenetetrahydrofolate reductase (MTHFR)] implied in oxidant reactions and inflammation [105]. Some of these genes have also been related to insulin resistance or diabetes.

#### 5.4.3. MicroRNAs

Nuts may modulate the expression of genes related to glucose metabolism through the mediatory effect of nutrients on microRNAs (miRNAs), defined as small non-coding RNAs with 20 to 25 nucleotides that post-transcriptionally and negatively regulate gene expression.

In the EPIRDERM Study, seven human circulating miRNAs were selected for analysis, that are considered widely related to glucose metabolism, insulin resistance status, prediabetes status, and biomarkers of T2D. The miRNA expression data showed that of the seven miRNAs studied, after pistachio intervention, circulating miRNA-192 and 375 expressions were significantly lower than in the control phase [78]. Furthermore, changes in the circulating miRNA-192 and miRNA-375, were positively associated with plasma glucose, insulin, and HOMA-IR, indicating that an increase in these miRNA levels mirror an increase in insulin resistance.

Similarly, in another trial involving 10 healthy women, 8 weeks of following a PUFA-enriched (achieved via daily intake of 30 g of almonds and walnuts) normocaloric diet resulted in significant modifications to several common miRNAs [106]. Specifically, the authors found that changes in circulating PUFAs were associated with changes of plasma miRNA-106a; changes in plasma miRNA-130b and miRNA-221 were associated with changes in plasma C-reactive protein, and changes in plasma miRNA-125a-5p was associated with changes in plasma fasting triglycerides and adiponectin.

#### 5.4.4. Metabolomics Modulation

Nutritional metabolomics is an emergent approach to obtaining deeper insights into diet–disease association that holds great promise in improving our understanding of the biological effects of nutritional factors and may help to identify potential novel markers of dietary intake and disease risk [107]. To date, a few studies have evaluated the impact of nut consumption on plasma or urinary metabolites [108].

Metabolomics has been used as an agnostic machine learning approach to identify plasma metabolites associated with walnut consumption using data from the PREvención con DIeta MEDiterránea (PREDIMED) study [109]. A metabolite profile including 19 metabolites (including lipids, purines, acylcarnitines, and certain amino acids) was associated with walnut consumption and with a lower risk of T2D incident in a Mediterranean population at high cardiovascular risk. These findings provide new insights into potential biological mechanisms explaining the effect of nut consumption on diabetes risk.

## 6. Current Strengths and Limitations

There are several strengths and limitations of the available evidence. Strengths, in general, include the relatively long follow-up duration of multiple years observed in the epidemiological studies and data from various countries allowing for potential generalizability of the findings. However, the cross-sectional and prospective cohort studies are limited by the inability to determine causation. A cross-sectional study design is also limited by the lack of ability to assess a temporal relationship between the exposure and the outcome. Additionally, most studies included in this review tended to obtain intake data via food frequency questionnaires (FFQs). FFQs have inherent weaknesses as they are subject to possible measurement error and recall bias [110]. Further, data from these FFQs were limited by the questions asked, as they often assessed a combination of nuts rather than a specific nut type and did not provide data on their preparation, such as whether the nuts were salted, spiced, roasted, or raw. Tree nuts and peanuts were often grouped together in the FFQs and were sometimes also combined within a question, including seeds and/or legumes. The doses of nut intake studied were also relatively low, even in the highest quantile of the analyses (with estimated median nut intakes ranging from 0 to 213 g/week), or were not sufficiently described, being presented as times or servings per day without an equivalent gram amount noted. Moreover, the majority of prospective cohorts evaluated nut intake at baseline as the dietary exposure; however, dietary habits may have changed over the course of the study follow-up period. This could have potentially resulted in misclassification of the exposure to nuts, hence biasing results and possibly explaining null associations observed with T2D. Of note, only a few large cohort studies have collected repeated measures of nuts and other dietary factors; these include the Nurses’ Health Study and Health Professionals’ Follow-up Study [18], in which diet was assessed every 4 years over 3–4 decades of follow-up. These repeated measures not only represent long-term dietary habits, but also can reduce measurement errors. Finally, as aforementioned, some prospective studies assessing the association between the frequency of nut consumption and diabetes risk have adjusted the analyses for body weight, which is an important determinant of diabetes and, thus, may lead to an attenuation of potential associations. Future cohort studies should carefully evaluate the role of body weight in mediating the association between total and different types of nut consumption and the risk of T2D.

While evidence from both acute and chronic RCTs in individuals with diabetes suggests nut consumption may improve glycemic control via reductions in fasting glucose and HbA1c, the effect still needs to be confirmed by updated SRMAs studying individuals with and without diabetes separately and without mixing interventions of whole nuts with nut oils or other extracts from nuts. It would also be useful to better understand which foods should be replaced with nuts in the diet for the most beneficial impact, as the current trials vary in this regard—some prescribe proportional reductions to all foods, and some suggest replacing for carbohydrate- or saturated fat-rich foods. In contrast, others provide no specific instructions on food replacement. Glycemic control assessment methods are also limited, and there is a lack of direct evidence from clamp studies or from Bergman´s minimal model, which may provide a greater understanding of metabolic regulation [111]. Moreover, there is limited trial evidence for the effect of pecans, pine nuts, Brazil nuts, macadamias, or peanuts in this area. However, since most nut types have similar nutrient profiles, the findings and associated recommendations are likely to be able to be extended to include all types of nuts.

In addition to the limitations to the currently available evidence, there are also a few potential barriers to nut consumption, such as nut-related allergies, cost, dental or swallowing issues, especially in older adults, and lack of knowledge of health benefits by health professionals [112]. This, in conjunction with the limited research evidence to support knowledge and potential recommendations, could potentially explain the relatively low intake levels of nuts by individuals worldwide [92].

## 7. Future Directions

Future research is needed to better elucidate the impact of nuts on the prevention and management of T2D. Given the current research limitations and limited epidemiological and clinical trial evidence available, there are several lines of research that could provide greater insight and better inform diabetes dietary guidelines.

Further investigation via prospective cohorts assessing the impact of nuts on diabetes incidence and pooled cohort analyses needs to be undertaken. Pooling data from currently conducted prospective cohorts may provide a relatively cost-effective and informative real-world way to explore the possible role of nuts in diabetes prevention and complications. Additionally, more studies in individuals with T2D are needed to demonstrate the impact of nuts on glycemic control (e.g., HbA1c, etc.). To determine a possible causal effect, conducting larger and longer RCTs (such as a multicentre RCT) evaluating markers of glycemic control as primary endpoints is needed in order to expand current knowledge to assess the effect of nuts on diabetes prevention in high-risk participants. Acutely, insulin sensitivity analysis testing the effect of nut consumption, such as replacing carbohydrates, using the Bergman Minimal Model of glucose regulation and clamps would aid in increasing the strength of available evidence.

Further explorations related to metabolomic and metagenomic signatures of nut consumption in clinical trials and assessing the association in long-term cohort studies of diabetes incidence and complications would additionally provide greater insights into a potential diet (nut)–disease (diabetes) association. Then a range of mechanistic molecular biological studies may be justified when a clear phenomenon, such as reduced insulin resistance and improved diabetes control, has been established.

## 8. Conclusions

Of the limited evidence currently available, overall findings suggest higher nut consumption may have beneficial effects on diabetes prevention and management. In particular, some but not all large cohort studies have found that higher consumption of total nuts, walnuts, and peanuts was significantly associated with a lower risk of T2D. Moreover, inclusion of nuts in the diets of individuals may have a beneficial effect on glycemic control and lower the risk of cardiovascular disease and mortality in those with T2D. In individuals with T2D, specifically, acute studies have demonstrated reductions in postprandial glucose levels, and long-term trials have indicated modest positive effects on blood glucose control, as shown by reductions in HbA1c and fasting blood glucose. Mechanistic pathways provide further promise for the potential role nut consumption may have in diabetes prevention and management. Despite all the potential diabetes-related health benefits nuts may pose, current evidence is not definitive, and there remains much opportunity for future research to address present weaknesses and limited data in this field to provide more conclusive evidence on the role of nuts in the prevention and management of diabetes.

## Figures and Tables

**Table 1:** Summary of findings related to nuts and diabetes related prevention and management.

Variables	Finding ^1^	Level of Evidence ^2^	Reference
***Epidemiological Evidence***			
Fasting blood glucose	↓	+	[11]
Plasma insulin	↓	+	
HOMA-IR	↓	+	[11,12]
HOMA-B	↓	+	[11]
HbA1c	↓	+	
OGTT	↓	+	
Diabetes incidence	↓/↔	+	[13,14]
Diabetes prevalence	↓/↔	+	[13,15,16,17]
CVD incidence in participants with T2D	↓	+	[18]
Diabetes mortality	↓	++	[19]
***Clinical Trial Evidence***			
***Acute Trial Evidence***			
*In participants free of T2D:*			
Postprandial glycemia	↓	++	[20,21,22,23,24,25,26]
Postprandial insulinemia	↓/↔	+	[21,27,28]
*In participants with T2D:*			
Postprandial glycemia	↓	+	[21,23,29,30]
Postprandial insulinemia	↓/↔	+	[21,29]
Glucose metabolic clearance rate	↑	+	[29]
***Longer-term Trial Evidence***			
*In participants free of T2D at baseline:*			
Diabetes incidence	↓/↔	+	[31,32,33]
*In participants with T2D at baseline:*			
Fasting glucose	↓	+	[30,34,35,36,37,38,39]
Fasting insulin	↔	+	
HbA1c	↓	+	
HOMA-IR	↔	+	
*In participants with/without T2D at baseline:*			
Fasting glucose	↔	+	[40]
Fasting insulin	↓	+	
HbA1c	↔	+	
HOMA-IR	↓	+	

Abbreviations: CVD, cardiovascular disease; HbA1c, glycated hemoglobin; HOMA, homoeostasis model assessment; IR, insulin resistance; OGTT, oral glucose tolerance test; T2D, type 2 diabetes mellitus. ^1^ Findings are based on the authors’ review, and assessment of the noted literature, and hence could present some subjectivity. In general: ↓, majority of evidence indicated a decrease; ↑, majority of evidence indicated an increase; ↔, majority of evidence indicated no change observed; ↓/↔, majority of evidence was split between showing a decrease or no effect on the outcome. Where “majority of evidence” refers to the entirety of the evidence, if a relevant systematic review and meta-analysis was conducted these findings were used as the basis of this determination. ^2^ Level of Evidence is based on the authors’ review and assessment and, hence, could present with some subjectivity. In general: +, limited and/or inconsistent evidence from few studies in the denoted type of study design; ++, consistent evidence in several studies in the denoted type of study design.

## Data Availability

Not applicable.
